# Investigating syntactic priming cumulative effects in MT-human interaction

**DOI:** 10.12688/openreseurope.13902.1

**Published:** 2021-08-12

**Authors:** Natália Resende

**Affiliations:** 1 School of Computing, Adapt Centre, Dublin City University, Dublin, Ireland

**Keywords:** Google Translate, Portuguese, English, Language Learning, SyntacticPriming, Machine Translation

## Abstract

**Background**: A question that deserves to be explored is whether the interaction between English language learners and the popular Google neural machine translation (GNMT) system could result in learning and increased production of a challenging syntactic structure in English that differs in word order between speakers first language and second language.

**Methods**: In this paper, we shed light on this issue by testing 30 Brazilian Portuguese L2 English speakers in order to investigate whether they tend to describe an image in English with a relation of possession between nouns using a prepositional noun phrase (e.g. *the cover of the book is red*) or re-use the alternative syntactic structure seen in the output of the GNMT (e.g. *the book cover is red*), thus manifesting syntactic priming effects. In addition, we tested whether, after continuous exposure to the challenging L2 structure through Google Translate output, speakers would adapt to that structure in the course of the experiment, thus manifesting syntactic priming cumulative effects.

**Results**: Our results show a robust syntactic priming effect as well as a robust cumulative effect.

**Conclusions**: These results suggest that GNMT can influence L2 English learners linguistic behaviour and that L2 English learners unconsciously learn from the GNMT with continuous exposure to its output.

## Plain language summary

In this research, we aim to investigate whether web-based popular translation tools such as google neural machine translation (GNMT) are capable of influencing the language behaviour of the English students that use them for learning purposes. We also investigate whether English students learn from the output of the systems and how this learning takes place, i.e., whether consciously or unconsciously.

## Introduction

Over the last few years, the output quality of machine translation (MT) has improved considerably thanks to the emergence of the neural machine translation (NMT) systems. The NMT systems represent a new type of statistical engine that uses neural networks algorithms originally inspired by the functioning of the biological brain ^1^. With this new paradigm, the NMT systems began to “learn” from the data through the association of patterns, confirming hypotheses in the field of psycholinguistics, according to which the brain organizes language in a series of neural networks that operate based on the association of frequent patterns ^2^.

In addition to improvements in translation quality, MT technology has also improved users’ access to those systems. Nowadays, users can choose to access an MT through a mobile application or a web browser. Moreover, MT users can choose between translation modalities, such as speech, text or image translation. These advances in translation quality coupled with easier access have led NMT systems to become the heroes of intercultural environments. The last world cup in Russia, for example, was considered the *Google Translate world cup*. Google reported a 30% increase in user access to the Google Translate (GNMT) system application during the tournament in Russia, especially translating from Spanish into Russian using the voice translation mode ^3^. This event shows that the way people are engaging with MT technology is changing. Instead of relying traditionally on tourist booklets for basic phrases, questions and expressions to communicate in the country’s native language, the ease of access to the tools has led tourists to use MT as a tool for quick translation of simple sentences for basic communication in Russian.

Although it can be thought that advances in the development of even more accurate translation systems may compromise interest in language learning, research in the field of computer assisted language learning (CALL) shows that, contrary to these concerns, MT systems are being used as tools supporting second language learning ^4^. The study by Garcia and Pena ^5^ illustrates this trend. The researchers have shown that free online MT systems help students with little command of the second language (L2) to improve their writing skills. With MT mediation, the study has shown that L2 learners communicated better, i.e., with higher quality, in the L2. Results have also revealed that the lower the L2 level, the greater the difference between the number of words composed using an MT and the number of those written directly into L2. Another study ^6^ with undergraduate students from Duke University has shown that, even against the advice of their teachers, students admitted to using the GNMT in their foreign language tasks.

Considering the current scenario in which, on one hand, we see changes in the way people are engaging with MT technology and, on the other hand, its use for language learning purposes, a question that deserves to be explored is related to the role the popular GNMT system plays in the cognitive processing of a second language, in particular, English as a second language (L2 English) due the global spread of interest in English learning ^7^ and its emergence as *lingua franca* ^8^.

In this paper, we hypothesize that GNMT output is facilitating L2 English learners to move from more semantic to syntactic processing, especially those in the early stages of language learning. It has already been observed that less proficient learners rely more on the lexical memory of isolated words, so they encounter difficulties and less automaticity in language tasks that involve the proper combination of words in sentences ^9^. Therefore, it is at this stage and for that purpose that translation tools represent an important source of support. Thus, it may be that, when using GNMT as a tool to support sentence construction in English, the output of GNMT may not only facilitate learners’ awareness of the differences in word order between their first language and the target language but also influence students to unconsciously produce sentences in the target language using the structure seen in the MT output. While in the field of second language acquisition (SLA) the interaction hypothesis ^10^ claims that human-human interaction facilitates second language development as it raises learners’ awareness of language forms, it may be that human-MT interaction has the potential to raise the same awareness in the target language and enable learners to learn challenging structures and modify grammatically incorrect syntactic structures for better communication.

### Rationale for the present study

The goal of the present study is to investigate whether the interaction between L2 English learners and GNMT can result in the learning of structures that are challenging for L2 English learners to process. It is also our aim to investigate whether any learning trends that can possibly be observed are of implicit (unconscious) or explicit (conscious) nature.

In order to accomplish this paper’s goal, we employed a syntactic priming study which is an experimental methodology widely used in the field of psycholinguistics to address issues related to language syntactic processing ^11– 14^. This experimental paradigm will allow us to identify learning trends in our data by examining whether L2 English speakers manifest so-called *cumulative effects*.

Cumulative effects are associated with the implicit learning account of syntactic priming ^11, 13^. According to this view, syntactic learning is of unconscious nature and it emerges as a result of continuous exposure to a certain structure. Although in the syntactic priming literature there is a controversy concerning the nature of the repetition phenomenon,i.e., whether it is of implicit (i.e. unconscious) or explicit (i.e. conscious) nature, a number of studies (e.g. 15– 17) have demonstrated that continuous exposure to a certain syntactic structure across trials leads to long-lasting adaptation (or learning) within the language production system. In other words, increasing the subjects’ experience with a certain structure affects the magnitude of the syntactic priming effect.

Following Shin and Christianson (2012), we adopted a pre-test-priming design as it enabled us to study the influence of the GNMT on participants’ performance in a picture description priming task as compared to the pre-test baseline. We have chosen this methodology because, as we will see in the next section, its ecological validity to study both L2 learning and human language behaviour when interacting with artificial partners has been already tested by previous studies ^16, 18– 20^.

In our previous paper ^19^, we tested 20 Brazilian Portuguese L2 English students to check whether, after Google translating Portuguese sentences expressing a relationship of possession between nouns (e.g. *A janela do quarto está fechada* - “the bedroom window is closed”) into English, they would describe images in English using the same syntactic structure previously seen in the GNMT output (which differs in word order from learners’ first language), that is, whether they would be primed by the MT output or whether they would choose a syntactic alternative that resembles the most common syntactic alternative in Portuguese to convey the same idea. We observed a robust priming effect suggesting that participants were influenced by GNMT syntactic alternative when describing images in English. In a subsequent study ^21^, we introduced a post-test phase the day after the priming test phase, in which participants were asked to describe the same images they described in the baseline pre-test phase (without using GNMT). With this design, we were able to observe whether the priming effect lasted from the priming session until the next day. Results showed that participants tended to describe the images using the syntactic alternative they had seen in the GNMT output the day before, rather than the syntactic alternative that resembled the word order in their native language. These results suggest that participants learned a challenging structure in English through the GNMT output. However, it was unclear to us how this syntactic alignment with the output of GNMT emerged. Specifically, it was unclear whether the priming effect observed in our two previous studies was the result of an implicit learning process triggered by the system output during the experimental session or the result of a learning effect that emerged from the first priming trials presented to the participants. Thus, in this paper, we tested whether the probability of producing the GNMT syntactic alternative when describing images in English increased over time as the experiment proceeded, thus manifesting cumulative effects with the participants’ continued exposure to this structure, or the priming effect was triggered by the GNMT output from the beginning of the experimental session onwards. In addition, we investigate whether any cumulative effect observed will vary as a function of participants’ English proficiency.

In the present follow-up study, we address the following research questions:

- **RQ1** *Can Google translate facilitate the processing of more challenging structures in the second language?*
- **RQ2** *If RQ1 is true, can learning emerge from the interaction between users and the GNMT system through continuous exposure to that structure via MT output?*
- **RQ3** *If RQ2 is true, does this learning vary as a function of participants’ English proficiency?*

As in the two previous studies, we will examine participants’ language behaviour when producing an English noun phrase expressing a relationship of possession between nouns (e.g. *the cutlery handles are colourful* or *the handles of the cutlery are colourful*). We focused on this structure as this type of noun phrase varies across the participants’ native and non-native languages.

In Portuguese, only one syntactic alternative exists to represent a relationship of possession between nouns. The relationship is always encoded in the preposition *do* (de + o) or *da* (de + a) (e.g. *a mesa do escritório está cheia* or *a porta da casa está fechada*). However, in English, this relationship can be represented using either a prepositional noun phrase (PNP), which follows the same word order as in Portuguese (e.g. *the table of the office is full*) or a non-prepositional noun phrase (NP) (e.g. *the office table is full*), which differs from Portuguese in word order. This allows us to identify whether syntactic priming by the GNMT output can lead Portuguese speakers to produce NP structures more frequently in English, which is an unfamiliar and more complex structure for these L2 speakers to process in English.

Before describing our methodology in detail, we review experimental syntactic priming evidence to demonstrate that the syntactic priming rationale represents an ecologically valid approach to address issues involving L2 learning as well as issues involving the interaction between humans and artificial systems.

## Background

### Cumulative effects in syntactic priming experiments

*Syntactic priming*, also known in the literature as *structural priming* or *syntactic alignment* (for a complete review on the terminology see 22), can be defined as the tendency of speakers to repeat a syntactic structure previously processed ^13^. It also refers to the facilitation of syntactic processing when a syntactic structure is repeated across consecutive sentences ^23, 24^.

The first laboratory study to investigate the syntactic repetition effect dates back to the 80’s ^13^. In this first study, participants were exposed to transitive sentences in either active (e.g. *One of the fans punched the referee*) or passive (e.g. *The referee was punched by one of the fans*) forms and to dative sentences in either prepositional-object (e.g. *A rock climber sold some cocaine to an undercover agent*) or double-object (e.g. *A rock climber sold an undercover agent some cocaine*) forms. After requesting participants repeat the sentences out loud, researchers asked participants to describe images depicting transitive scenes unrelated to the repeated sentences. The results of this study revealed that participants tended to describe the images using the same structure they had previously produced, i.e., they described the images using passive sentences if they had previously produced passive sentences, double-object sentences if they had previously produced double-object sentences, and so on. This seminal study has paved the way for the emergence of a number of other studies using the same methodological paradigm to investigate the repetition effect. Such interest is based on the assumption that investigating the repetition effect of syntactic structures could help researchers to understand aspects of the nature of human syntactic knowledge, the mechanisms underlying this knowledge as well as aspects related to its learning and development.

Over the few last decades, the effort devoted to research on the syntactic priming effect has brought some insights concerning the nature of the relationship between lexical and syntactic processing. Research has revealed, for instance, that the syntactic priming effect is more frequently driven by the least preferable syntactic structure. A number of studies (e.g. 16, 17, 25, 23) have shown that passive constructions, which are less frequent in English and Dutch, prime more than the highly frequent active constructions. Similarly, the less common dative constructions in English and Dutch prime more than the frequent prepositional double-object constructions. Such an effect, known in the literature as the *inverse preference effect*, is explained by researchers as a consequence of the cognitive mechanism reorganization driven by the surprise of an unexpected structure ^17, 23, 26^ as well as by the so-called *cumulative effect* or *accommodation effect*.

The *cumulative effect* occurs when the priming probability (i.e. repetition of syntactic structures previously processed) increases with one’s continuous exposure to that syntactic structure over time ^17^. Studies reporting cumulative effects show, therefore, that participants adapt their language behaviour to the least preferable structure throughout the entire experimental session.

Some studies (e.g. 11, 27) revealed that, when participants were more frequently exposed to one of two structures, the production of the other alternative structure was reduced. For instance, if prepositional datives were more frequently used by participants after repeated exposure to prepositional datives, the double-object datives become less frequently used.

In a study analysing priming effects for word order of auxiliary verbs and the past participle in Dutch subordinate clauses ^27^, researchers found that participants tended to keep the same word order as the prime sentence in the target sentence. In addition, this experiment has shown a cumulative effect as the use of the least preferred structure (the auxiliary-final structure) became the more preferred in later trials of the experiment compared to the earlier trials, replicating results reported in their previous studies ^28^.

Another source of evidence comes from the corpus of spontaneous speech ^17^. The researchers counted the number of primes that were either comprehended or produced by the same speaker up to the point of the target. Results showed that the more passives the speaker had previously produced in the conversation, the more likely they were to produce another passive in the subsequent utterance. They also found that the more actives speakers produced previously, the less likely they were to produce a passive. Again, these observations suggest that continuous exposure of an infrequent structure increases the probability of that structure being produced later.

In a study testing patients with Korsakoff’s syndrome, cumulative effects were also found ^29^. Although the aim of the study was to investigate the memory system that supports syntactic priming effects, the researchers analysed learning trends in the data by calculating the proportion of passives out of the total number of transitive responses produced in the target trials before the current target trial. Results showed that the more passives produced, the stronger the effect, revealing a learning effect of priming in patients with Korsakoff’s syndrome. Similarly, in the L2 literature, studies investigating L2 learning through syntactic priming have demonstrated that continuous exposure to a challenging structure to process in the L2 leads to the learning of that structure ^30– 32^.

Syntactic priming is also manifested as facilitation in syntactic processing. A number of studies (e.g. 23, 33– 35) have shown that participants exposed to a certain syntactic structure tend to process the subsequent sentence with the same structure faster, suggesting that the prime sentence plays a key role in the amount of language resources recruited to produce a sentence. The facilitatory characteristic of the syntactic priming effect has been observed not only in speech production contexts but also in contexts of sentence comprehension.

As syntactic priming is a well-studied phenomenon and can inform researchers about various aspects of syntactic processes, other fields of research have adopted the syntactic priming methodological paradigm to address a variety of research questions related to language processing. In the field of second language acquisition (SLA), syntactic priming methods have been used to investigate the differences and similarities in syntactic processing between the first (L1) and second languages (L2) as well as the impact of the priming effect in the acquisition and processing of a second language ^20^. Studies on human-computer interaction (HCI) have also benefited from the syntactic priming methodological paradigm when investigating linguistic behaviour in speech-based interactions between humans and intelligent systems ^15, 16, 18, 36^. Below we review studies using the rationale of the syntactic priming methodological paradigm to investigate the influence of intelligent systems on language behaviour and to address issues related to syntactic processing in a second language.

### Syntactic priming in HCI

Although syntactic priming effects have been largely observed in monolingual studies in different languages, using different methodological paradigms and different syntactic structures, linguistic alignment has also been demonstrated to occur between humans and computers and artificial systems ^16, 37^. Research ^36^ has found, for instance, that children talking to computer partners spontaneously adapt several basic acoustic and prosodic features of their speech by 10–50%, with the largest adaptations involving utterance pause structure and amplitude. Prosodic alignment has also been demonstrated to occur between humans and computers ^38^. Moreover, work has found that humans align their lexical choice and gesture handedness in similar ways when interacting with human partners and virtual partners ^39^.

Regarding syntactic alignment, in human-computer speech-based interactions syntactic priming has been found to occur for both dative structures (e.g. *give the waitress an apple* vs. *give the apple to the waitress*) and noun phrase structures (e.g. *a purple circle* vs. *a circle that is purple*) evidencing that a computer system can also influence a speakers’ grammatical choices in speech-based interactions ^18^. More recently, virtual reality studies have shown syntactic alignment between humans and computer avatars ^16^. The effect has been observed for passives and actives, although the priming effect was stronger for passives than for actives. In a naturalistic experiment with the dialogue system *Let’s Go!*, as in laboratory experiments, users adapt to the system’s lexical and syntactic choices ^40^.

All these pieces of evidence in the literature suggest that, even though aware of the artificial nature of the partners, in a communication context, humans tend to consider artificial systems as social actors and, when interacting with them, apply human communication strategies.

### Syntactic priming in the field of SLA

Syntactic priming experiments have also been proven to be useful to study second language learning because the syntactic repetition effect is interpreted as a learning process that occurs during the mapping between message form and its meaning, which leads to the subsequent use of the sentence form ^41^.

In the SLA literature, the syntactic priming experiments follow the same rationale as the syntactic priming experiments within the L1 literature; that is, researchers in the field of SLA test whether a speaker’s production is influenced by the structure that was present in the preceding discourse despite the availability of another accepted structure to convey the same meaning.

A seminal study was carried out by Hartsuiker, Pickering and Veltkamp ^42^. They investigated whether Spanish L2 English bilinguals would describe pictures in English using passive structures after they listened to passives structures in Spanish or whether they would choose the active structure. In this experiment, researchers found cross-linguistic syntactic priming as participants produced more passive picture descriptions in English after they had just heard a Spanish passive sentence. This work has shown that structural priming plays a beneficial role in L2 development in the production of active and passive structures in English ^30^.

Different structures have also been tested such as *Wh-* question production in L2 English ^32, 43, 44^. In these studies, researchers tested whether interacting with more advanced English learners would improve learners’ performance in producing *Wh-* questions with the supplied auxiliary verb (e.g. *why do people buy products?*) instead of the interlanguage form in which the obligatory auxiliary verb is missing (e.g. *why people buy products?*). The researchers assumed that hearing or producing the advanced *Wh-* question would function as a template for the production of the subsequent use of that form as opposed to the less advanced form. The results of both experiments showed that syntactic priming played a role in the development of *Wh-* question formation.

Syntactic priming for prepositional-object datives has also been tested in the L2 ^31, 44^. Results have shown that participants produced more prepositional-object datives when they had previously heard or produced the prepositional-object structure themselves than when they had not.

A study of Korean L2 English learners ^20^ investigated whether structural priming improves performance in producing complex, double-object dative (e.g. *The boy is handing the singer a guitar*) and simple, separated phrasal-verb structures (e.g. *The man is putting the fire out*), which are structures that Korean L2 English learners have difficulties in producing. Results showed that syntactic priming improved complex dative production and this improvement was observed to persist over time.

More recently, syntactic priming has been investigated in adverb–verb–subject structures (e.g. *In the winter, jack wears a jacket*) vs. subject–verb–adverb order structures (e.g. *Jack wears a jacket in the winter*) among intermediate English–German second language learners. Participants exhibited comparable short-term priming for adverb-first word order.

From the literature presented above, it can be noted that syntactic priming represents a fruitful methodological approach to study both syntactic processing in L1 and L2, as well as to investigate the impact of machines on human linguistic behaviour. However, unlike the present study, the studies reported above did not address the issue of English students’ internal cognitive processes related to implicit learning via structural priming emerging in HCI. Nor has this earlier work examined the effects of structural priming from language comprehension (reading) to production (speaking) through a cross-linguistic task (translation). Therefore, in this paper we intend to contribute to the HCI, MT and SLA fields by carrying out an experiment that involves all these issues. We believe that the present study will bring important insights to the question of whether peoples’ grammatical choices can be influenced by the MT systems’ grammatical choices and whether this experience could result in (implicit) second language learning.

## Methods

In this paper, using a syntactic priming paradigm we investigate cumulative effects with the objective of detecting learning trends elicited by GNMT in the processing of NP in L2 English learners. The experiment was constructed to be carried out in two phases: pre-test phase and priming phase. We will analyse whether participants will change their language behaviour from the pre-test phase to the priming phase. Thus, we considered the pre-test our baseline as, in this phase, participants were not influenced by the MT output.

The rationale behind our experiment is:

- If, in the priming phase, participants describe the images in English using the same structure previously seen in the GNMT output more frequently than the structures used in the pre-test phase (which does not involve any interaction with GNMT), then our results suggest that GNMT is capable of influencing participants’ grammatical choices.
- If we observe that the use of the challenge structure increases in the later trials with continuous exposure to the same structure leading participants to adapt to that structure in the course of the experiment, our data suggest that a implicit learning process took place in the course of the experiment as every instance of syntactic structure updates the speaker’s knowledge of that structure.

According to some researchers ^45^, learning occurs because speakers adapt to the context with the aim of reducing errors and uses all the information available to them for this purpose. Therefore, we hypothesise that will we see a priming effect emerging in the course of the experiment, suggesting that GNMT is playing a role in the learning of English syntactic structures as well as in shaping speakers’ syntactic processing.

### Ethics

The study was conducted according to the guidelines of the Declaration of Helsinki, and approved by the Ethics Committee of Dublin City University (protocol code: DCUREC/2019/110, date of approval: 21 June 2019.) Written informed consent in which participants consented to voluntarily take part in the study and have their demographic data published in journals and elsewhere was obtained from all subjects involved in the study.

### Participants

We analysed data from 30 volunteer Brazilian Portuguese L2 English speakers (10 men, mean age=35.7 - sd=5.3). Participants were recruited through posts on Facebook groups of Brazilians living in Dublin and word-of-mouth recommendations. All participants were requested to read a plain language statement and sign the informed consent form to take part in the experiment. The inclusion criteria to take part in the study was to be a native speaker of Brazilian Portuguese, to live in Dublin at the time of the data collection, to have used GNMT as a tool supporting spoken English and to be at intermediate or advanced English levels. In order to test participants’ English proficiency levels we asked them to complete the General English test online with 25 questions immediately after completing the the pre-test and priming test phases. Participants were classified at the basic level (level A2) with scores ranging from 2 to 13; pre-intermediate level (B1) scores from 14 to 17; intermediate level (B2) scores from 18 to 19; advanced level scores from 20 to 22 and proficient level scores from 23 to 25. The mean score of the participants included in the experiment was 14.1 (SD=4.7) or level B1). In return for taking part in the experiment, all participants received a €10 voucher.

### Materials

***Baseline pre-test.*** For the baseline pre-test, we created a total of 26 sentences in Portuguese and we selected 26 images depicting those sentences from an online image repository ^46^.

**Figure 1.**
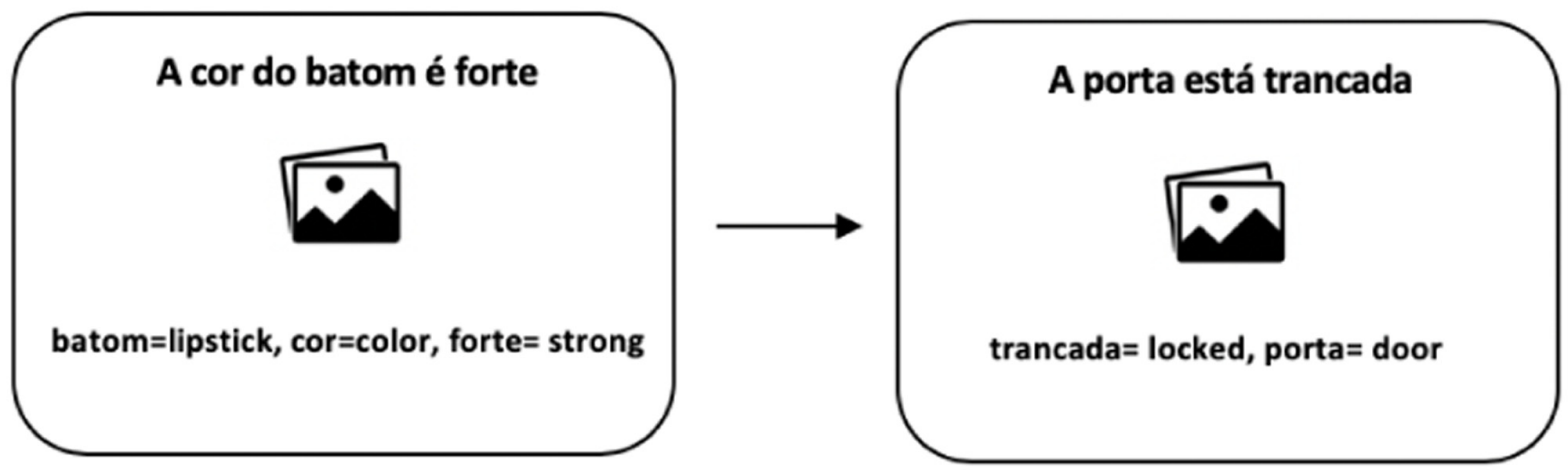
Example of trials presented in the baseline pre-test. Participants translated the sentences depicting images out loud using the words provided below each image left figure example of trial and right figure example of filler trial.

The experimental trials consisted of 20 sentences and images depicting those sentences. The sentences expressed a relationship of possession between nouns and they were composed of a noun phrase + to be verb + complement (e.g. *a janela do escritório está quebrada*, which could be translated by participants using either a prepositional noun phrase structure (PNP) such as *The window of the office is broken* or a noun phrase structure (NP) such as *The office window is broken*. The remaining six trials (30% of the experimental trials) were filler trials which were constructed with sentences composed of subject + to be verb + complement (e.g. *A porta está trancada - ”the door is locked”*) and images depicting those sentences. Figure 1 illustrates the trials presented to participants in the baseline pre-test phase.

**Figure 2.**
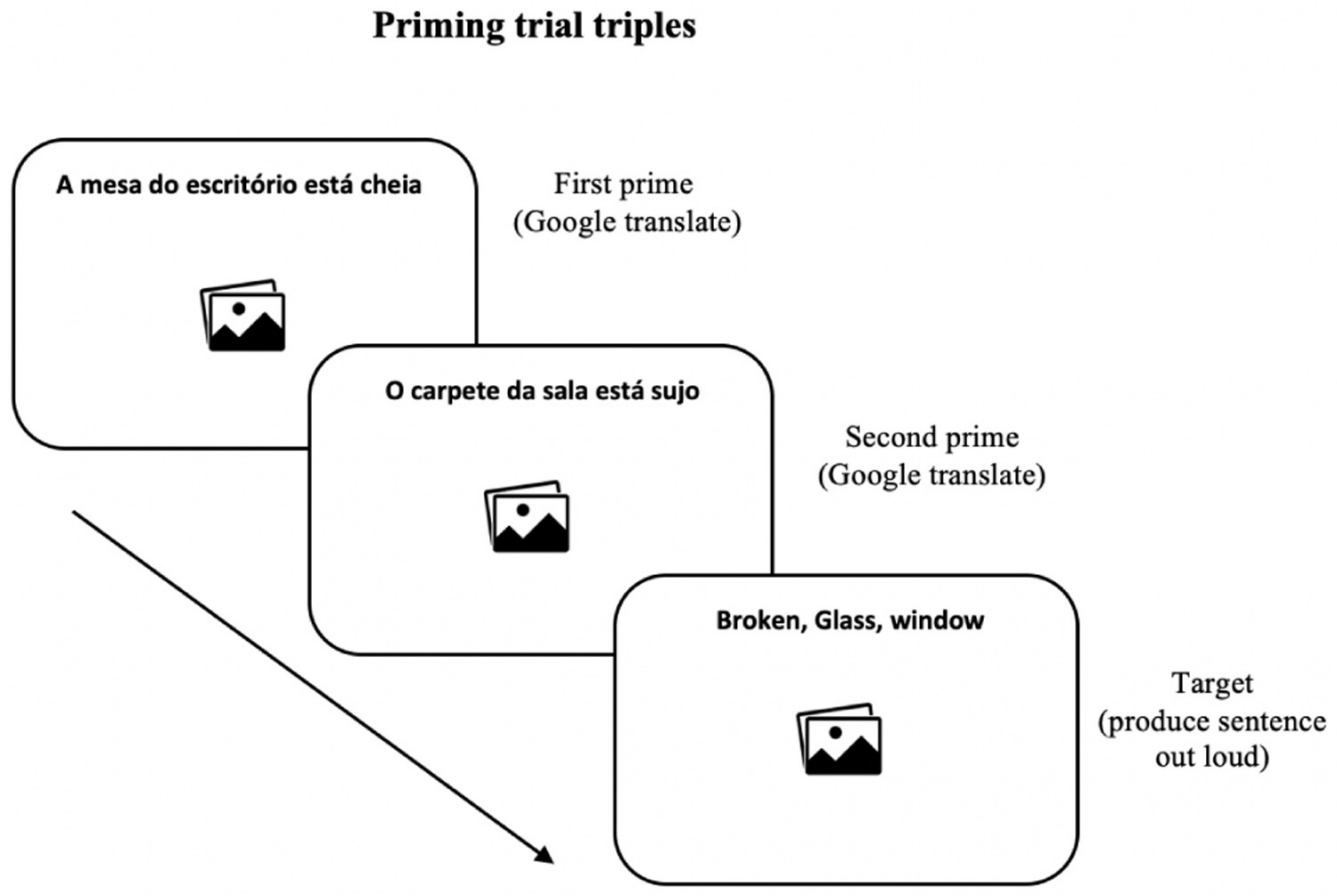
Example of trial presented in the priming test. Participants Google translated the sentences of the first and second primes. In the target item, they were instructed to describe the images out loud using the words provided above each image.

**Figure 3.**
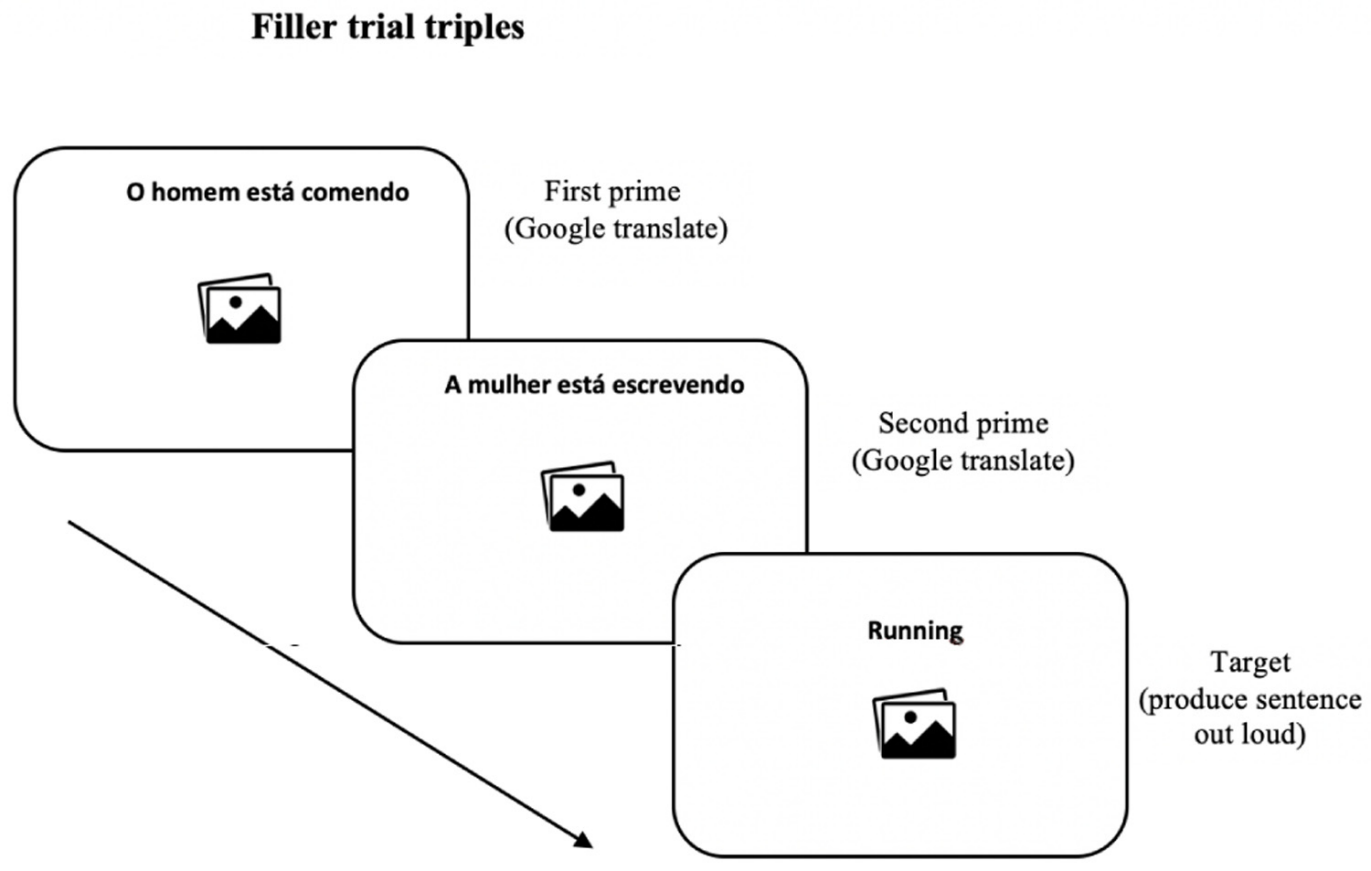
Example of trial presented as fillers. Participants Google translated the sentences of the first and second primes. In the target item, they were instructed to describe the images out loud using the verb provided above each image.

***Priming test.*** To construct the priming phase trials, we created a total of 26 prime-target triplets trials ^46^. As in the pre-test phase, 20 prime-target triplets out of 26 prime-target triplets were experimental trials while the remaining six prime-target triplets were filler trials (30% of the experimental trials). Figure 2 illustrates the 20 experimental trials presented to participants in the priming phase and Figure 3 illustrates the filler trials of the priming phase.

***The 26 prime-target triplets.*** The 20 prime-target triplets were composed of two prime items preceding a target item, thus we used 40 sentences and 40 images depicting those sentences to create the prime items and 20 sentences and 20 images to depicting those sentences to create the targets, totalling 60 sentences and images depicting those sentences.

To construct the six filler prime-target triplets we used 12 sentences and 12 images depicting those sentences to create the prime items and six sentences and six images depicting those sentences to create the targets, totalling 18 sentences and 18 images depicting those sentences ^46^. Thus, in total, the priming test phase used a total of 78 sentences and 78 images depicting those sentences (60 sentences and 60 images for the 20 prime-target triplets and 18 images and 18 sentences for the filler prime-target triplets).

The two prime items preceding the target of the 20 experimental trials (the 20 prime-target triplets) consisted of a sentence in Portuguese composed of a noun phrase expressing a relationship of possession between nouns + to be verb + complement (e.g. *A capa do livro é vermelha* - ”The book cover is red”), which were presented to participants to translate from Portuguese into English using a GNMT application on their own mobile device. The target consisted of an image and three English words (randomised across target trials) appearing above the images.

The two prime items preceding the target of the remaining six filler trials (the six prime-target triplets) consisted of sentences in Portuguese composed of a subject + an intransitive verb (e.g. *The man is writing*) presented to participants to translate from Portuguese into English using a GNMT application on their own mobile device. The target consisted of an image for description in English and an intransitive verb (e.g. *knitting*) appearing above the image.

### Procedures

Following Cowan *et al.* ^18^, the experimenter noted the syntactic structure (NP, PNP, ’S or Other) used by the 30 participants to describe the target images in English. The experimenter noted as “PNP” if the image was described by the participant using the English preposition ”of” (e.g. *the table of the office*); and as ”NP” if participant used a noun phrase a structure in English without the preposition in the correct word order (e.g. *The office table*) mirroring the GT output, and ”other” if they used a noun phase structure in the incorrect order (e.g. *the table office*), or the genitive case (e.g. *table’s office*) to express a relation of possession between nouns or for any other structure used (e.g. *the table in the office*) that differed from GT preferred syntactic alternative. These data were then considered the categorical dependent variable used for data analysises. All experimental sessions were audio recorded using *Quick Player* voice recorder and stimuli were presented in a computer screen (Macbook Pro with 13-inch Retina display screen) using *Psychopy* software version 3.0 which allowed us to randomise the stimuli set of both baseline and priming test phases across participants. The recordings of the sessions allowed a sanity check carried out by an independent blind rater to make sure that all data was correctly coded by the experimenter. The set of stimuli was presented visually and each participant performed the tasks individually in a silent room in the school of computing of the Dublin City University. Prior to the experimental sessions, the experimenter presented the instructions of the tasks to each participant while they appeared on the screen.

***Baseline pre-test phase.*** The baseline pre-test was presented to participants before the priming test. The order of presentation of the 20 trials and the six filler trials were randomised across participants. In this phase, participants were instructed to speak their translation of the sentences from Portuguese into English out loud using the English words presented at the bottom of the screen in order to avoid lexical retrieval issues in the L2 during the task (see Figure 1). All sentences could be translated from Portuguese into English using either a PNP structure or a NP structure.

***Priming test phase.*** The order of the presentation of the 26 prime-target triplets was randomised across participants. In the two prime items preceding the target item, participants were asked to translate the prime sentences above the images using the GT application on their own mobile device and repeat the translation out loud in order to trigger the syntactic priming effect ^11, 13, 20^.

Immediately after machine translating the prime sentences, participants were presented with the target item and were instructed to describe the image on the screen with a simple sentence using the three words appearing above the image (experimental trials) or the intransitive verb (filler trials). Participants were also instructed to avoid including words that were not on the computer screen and avoid describing the images using prepositions of location (such as *in*, *on*, *at*, etc). All images could be described using either a NP structure or a PNP structure.

 Two prime-target triplets in the two conditions (experimental and filler condition), not included in the main experiment, were used for participants’ training prior to the start of the experimental session.

### Coding and analysis

We analysed 40 data points per participant, totalling 1200 data points (40 × 30 = 1200). Data points from filler trials were not included in the analysis.

Participants’ responses for both baseline and priming tests were manually coded by the experimenter. We coded the baseline as ”1” if participants produced a NP structure, suggesting they were primed by the GT output and ”0” if participants produced a PNP structure. We coded the dependent variable *Prime* as ”yes” (1) if, in the target item, participants used the same structure previously seen in the GNMT output (in this case NP structures) to describe the images or ”no” (0) if participants described the image using a PNP structure or any other structure (such as *the office’s window is broken* or *the window in the office is broken*).

Analysis was carried out in R Studio version 1.1.423 ^47^ package lme4 ^48^. We used a logistic mixed effects maximal model including participants and items as random effects. The factors included as fixed effects were: *cumulative NP proportion* (continuous) to investigate learning trends in data, *English proficiency test score* (continuous) and *test type* (factorial). Following ^15, 16^, *cumulative NP proportion* was calculated as the proportion of NP structures out of all structures produced in the target items before the current target item. We included the *English proficiency test score* (continuous) and *text type* (factorial) with two levels: baseline or priming test. Factorial predictors were dummy coded (all means compared to a reference group) and all numeric predictors were centered.

### Predictions based on GT output

Before creating our experimental trials, we tested how GNMT would translate Portuguese sentences containing a relation of possession between nouns. We noticed that GNMT translated sentences from Portuguese into English using a NP structure far more frequently than a PNP structure ^46^. We also tested all the 40 sentences used to construct the prime items of the priming phase of the experiment and, again, we observed that GNMT translated them using a NP structure which is, as already mentioned, a more challenging syntactic alternative to Portuguese speakers due to differences in word order between learners’ first and second languages.

Because the English PNP structures with a relationship of possession between nouns resemble the noun phrase structure word order in Portuguese, we predict that, in the baseline pre-test, participants will produce more English PNP structures than NP structures, while in the priming phase, we will see a decrease in the use of this structure and, at the same time, an increase in the production of NP structures in the course of the experiment triggered by GNMT output.

In addition, based on a number of syntactic priming monolingual and bilingual studies showing learning trends in the data, we predict that the increase in production of NP structure will occur at later trials regardless of participants’ English proficiency levels.

## Results

Table 1 displays the percentage of structures produced by participants in both the pre-test and priming phases. As predicted, in the pre-test phase, participants produced 59.7% of constructions in PNP form while in the priming phase this percentage drops to 38.7% (a difference of 21%). In the priming phase, the average percentage production of NP structures increased by 26.7% and other structures (such as *the house’s door is yellow* or with the incorrect order of the noun phrase such as *the door house is yellow) decreased by 5.6%.

**Table 1.** Count and percentages of structures produced by 30 participants in the pre-test and priming phases with 20 trials each. PNP = preprositional noun phrase, NP = non-prepositional noun phrase.

Structures produced	Pretest	Priming
PNP	358 (59.7%)	232 (38.7%)
NP	168 (28%)	328 (54.7%)
Other	74 (12.3%)	40 (6.7%)
Total	600 (100%)	600 (100%)

Table 2 displays the model that best explains our data set. The intercept estimate is negative, meaning that NP structures in the priming phase were more frequent than in the pre-test baseline. The model shows a significant priming effect (p *<*0.001) as well as an effect of cumulative NP proportion (p = 0.01) to express the relationship of possession in the second language. This effect suggests that the amount of NP structures previously produced by the participants influenced the probability of producing NP structures in the subsequent utterances. This effect indicates a learning process taking place as NP production increases over time. This increase in the proportion of NP structures produced over time in the priming phase is demonstrated in Figure 5.

**Table 2.** Summary of the best mixed effects logit model for participants’ structure choices. Number of obs: 1200; groups: subjects = 30, items = 20. NP = non-prepositional noun phrase.

Random effects				
Group name	Variance	Std.Dev.		
Subject (Intercept)	5.767e-01	0.7593928		
Items (Intercept)	4.326e-10	0.0000208		
Fixed effects				
	Estimate	Std. Error	z value	Pr( *>*—z—)
Intercept (PNP structures or other)	-0.99042	0.18402	-5.382	7.35e-08 ***
English test grade	0.67375	0.17795	3.786	0.000153 ***
Cumulative NP structures	0.48263	0.10650	4.532	5.85e-06 ***
Priming test phase	113.027	0.16352	6.912	4.77e-12 ***
English test grade: cumulative	-0.06264	0.09733	-0.644	0.519863

To investigate if the learning process varied as a function of participants’ English proficiency levels, we tested the interaction between factors *cumulative NP proportion* and *English proficiency test score*. As we can see from Table 2, we did not find a significant interaction between these two factors (p=0.5), suggesting that, at all language proficiency levels, the amount of NP structures previously produced by the participants can influence the probability of producing NP structures in subsequent utterances.

**Figure 4.**
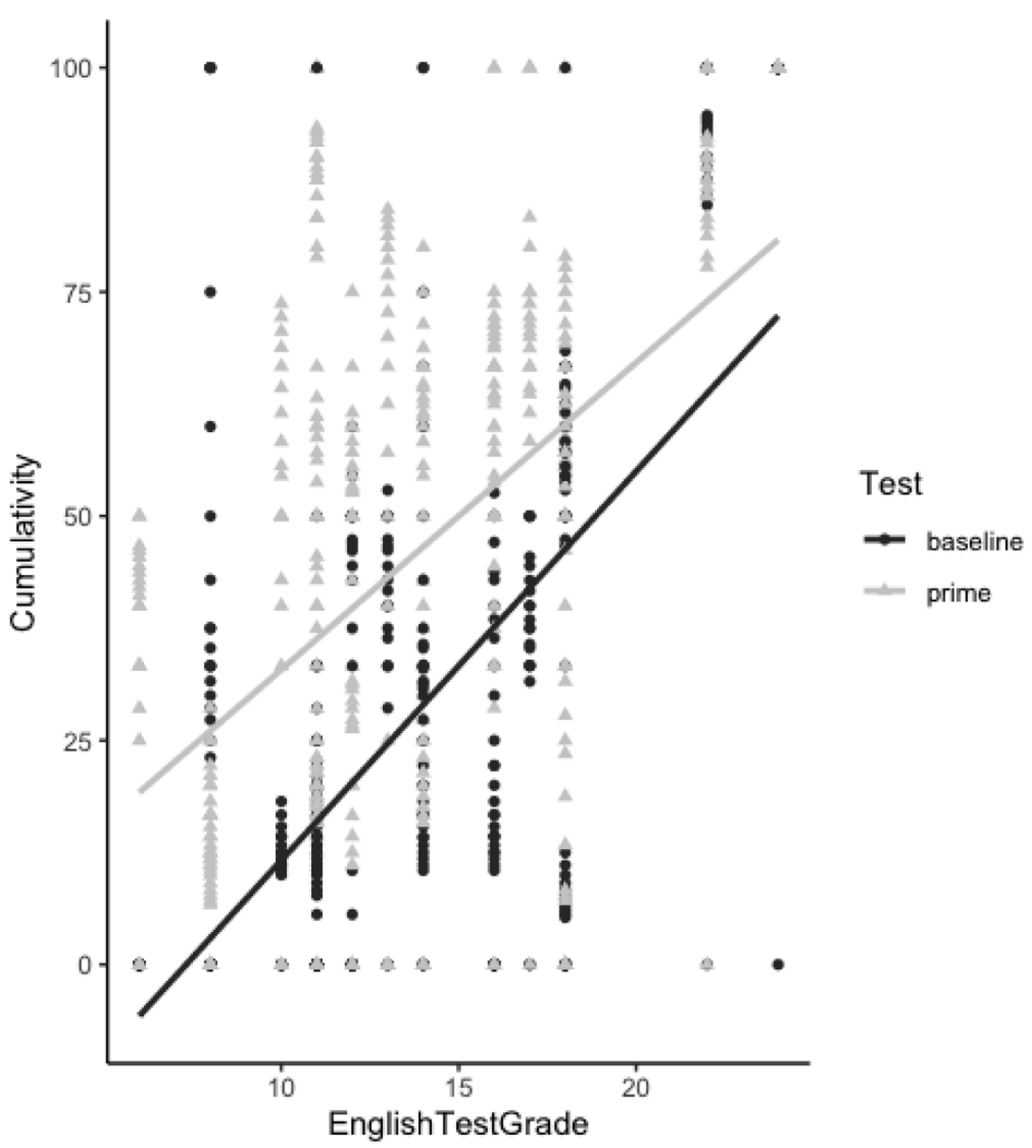
Cumulativity of non-prepositional noun phrase (NP) responses throughout experimental trials in both the baseline and priming phases. The proportion of NP responses produced increases over the course of the priming phase for all English proficiency levels (except for one outlier highly proficient participant who used the NP structure from the beginning until the end of the experimental session).

It is possible to observe in Figure 4 that this increase in the proportion of NP structures occurs at all language proficiency levels, except for a few highly proficient participants with an English test grade above 20 who produced NP structures from the beginning to the end of the experiment in both the baseline and priming phases. This therefore explains the lack of interaction between factor *English proficiency test score* and *cumulative NP proportion*, suggesting that learning and accommodation to a challenging structure can occur at all English proficiency levels.

**Figure 5.**
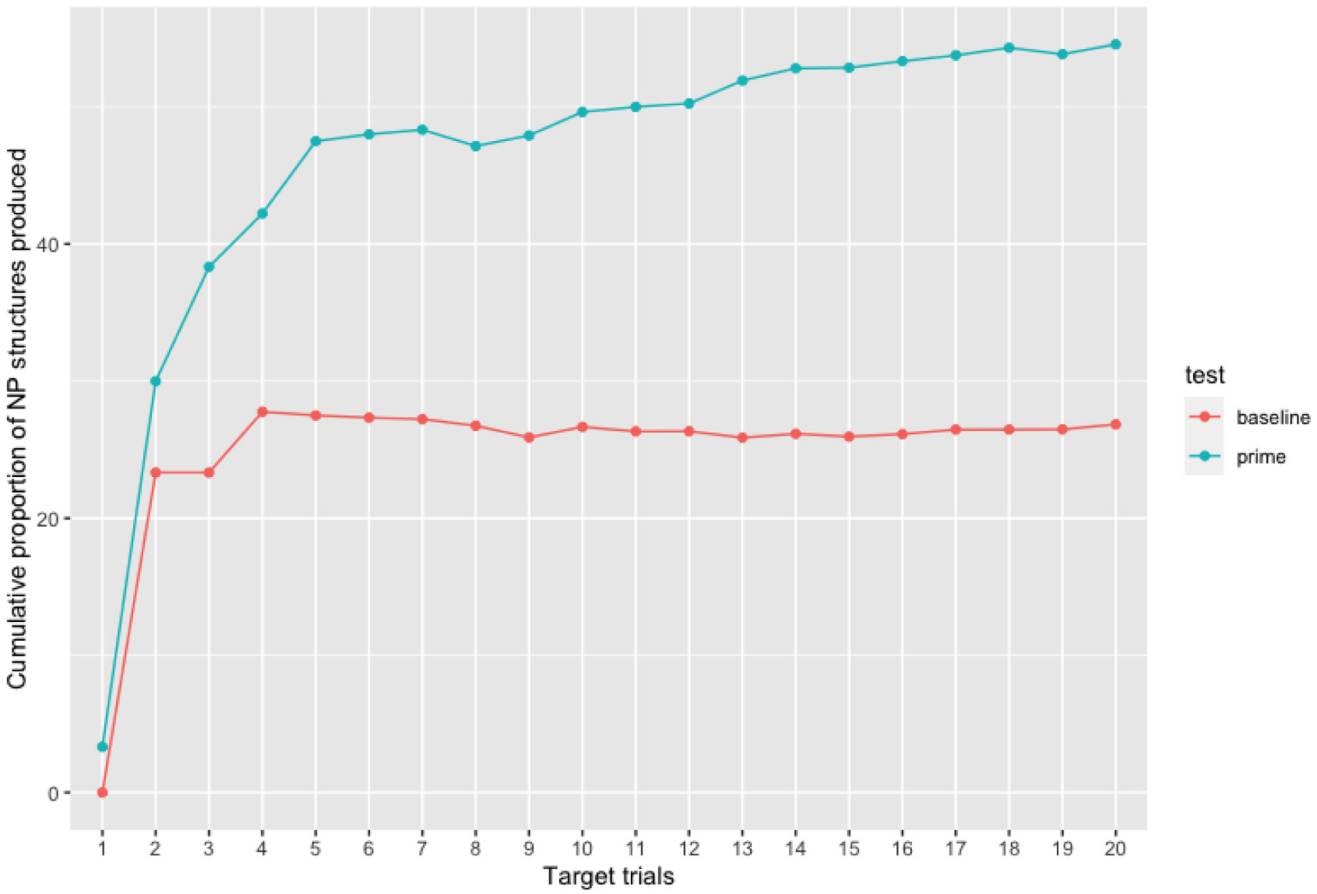
Cumulativity of non-prepositional noun phrase (NP) responses throughout experimental trials in both the baseline and priming phases. As compared to the baseline test, the proportion of NP responses produced increases over the course of the priming phase (from target 1 to target 20).

## Discussion and conclusion

Fully in line with our predictions, the effects observed in the present study clearly show that the GNMT system influences the processing of English syntax and that this influence is a consequence of cumulative exposure to the syntactic structure. Thus, our results answer in the affirmative RQ1 ( *Can Google translate facilitate the processing of more challenging structures in the second* language?) and RQ2 ( *Can learning emerge from the interaction between users and the GNMT system through continuous exposure to that structure via MT output?*). Such an influence is demonstrated in our results when a structure in English that is challenging for Brazilian Portuguese native speakers to process becomes more frequent in their subsequent speech after continuous exposure to the GNMT output with that structure. Thus, the syntactic priming effect observed in the present experiment is in line with previous results of studies investigating syntactic priming between humans and artificial systems ^15^ as well as in line with theoretical assumptions of surprisal and cumulativity ^17^, which predict that long-term priming results in a change in participants’ syntactic preference. The less frequent or the most challenging structure (in this case NP structures) becomes more frequent in the course of the experiment.

As we can observe from Table 1 and Figure 5, there is a difference in participants’ syntactic choice between the pre-test (without any influence of the MT output) and priming test. In the priming test phase, while the amount of PNP structures decreases, the amount of NP structures increases. Therefore, just like the surprisal and cumulativity theories claim, the learning mechanism was triggered by a less frequent structure and through continuous exposure to this structure the repetition effect increased. Therefore, our results suggest that an implicit learning mechanism can be activated in the second language by the syntactic repetition of an unusual structure in line with previous studies using a similar methodological paradigm ^20, 27, 28^. Regarding RQ3 ( *Does this learning vary as a function of participants’ English proficiency?*), our results suggest that this phenomenon can occur both at lower levels of English proficiency and at more advanced levels.

Our results can bring insights to the MT and HCI fields as they show that interaction with an MT system could help students internalise and unconsciously learn a difficult structure in the second language regardless of English proficiency levels. In addition, it is also curious to see that, regardless of the easily observable gaps in the translation quality of raw MT output, speakers trust Google translate enough to use the same syntactic structure seen in the MT output in their speech and learn from it. These results add to the growing body of evidence that speakers tend to align their linguistic behaviour with their conversational partners not only in in human-human interactions, but also in human-computer interactions in a cross-linguistic task.

We question, nevertheless, if the same effect could be observed in the interaction between a less popular MT system and humans. Within the syntactic priming literature, research has demonstrated that the social opinion one has of the interlocutor shapes the priming effect. Thus, it might be that less popular MT systems could fail to elicit syntactic priming effects or elicit less robust priming effects compared to GNMT. In future research we aim to address this question.

In future studies we also plan to investigate in depth the implicit nature of the learning effect observed here by testing if the cumulative effect emerges from different structures using less popular MT systems. If so, this would suggest that MT systems play a relevant role in the cognitive mechanism of sentence processing in the second language.

In summation, our results allow us to conclude that the syntactic priming paradigm represents an ecologically valid method to study MT-human interaction as well as the impact of MT in second language learning as our results replicate findings of a number of previous studies in the fields of HCI, SLA and Psycholinguistics. Thus, our study opens the possibility of addressing other research questions in MT-human interaction using the same methodological paradigm.

## Data availability

### Underlying data

Zenodo: Dataset MTrill project. https://doi.org/10.5281/zenodo.5138028 ^46^.

This project contains the following underlying data:

- data_30_subj cumulativity.csv (anonymised data for 30 participants)

### Extended data

Zenodo: Dataset MTrill project. https://doi.org/10.5281/zenodo.5138028 ^46^.

This project contains the following extended data:

- The 26 images and sentences used in the baseline phase (pre-test phase): 20 items used in the trials of interest and six items used as filler trials (in PDF format)
- The 78 images and sentences used in the Priming phase: 60 items used in the trials of interest and 18 items used as filler trials (in PDF format)
- translated_sentences_experiment_MTrill.xlsx (All sentences used to test Google Translation before running the experiments)
- stimuli_noun_phrase.docx and Noun_phrases_2.docx (Files with sentences used to test Google Translate prior choosing the sentences used in the experiment)
- MTrill.psyexp_version2.psyexp copy and images_prime_target_version2 copy.xlsx (Files used to run the experiment on Psychopy software)

Data are available under the terms of the Creative Commons Attribution International license (CC-BY 4.0).
