# Supplementary material for: Investigating syntactic priming cumulative effects in MT-human interaction
Source: Open Res Eur. 2023 Dec 13;1:93. Originally published 2021 Aug 12. [Version 2] doi: 10.12688/openreseurope.13902.2 (PMC10869954; doi:10.12688/openreseurope.13902.2)
Supplement: Peer review history

# Reviewer report: Reviewer response for version 2

**DOI:** 10.21956/openreseurope.18312.r36793

**Published:** 2024-02-15

**Authors:** Michael Carl, Longhui Zou

**Affiliations:** 1 Kent State University, Kent, OH, USA

I read the paper and found it to be nicely written and structured, complete review of literature, appropriate description of the experiment and impactful conclusion. 

 I think it can be indexed as is.

Is the study design appropriate and does the work have academic merit?

Partly

Is the work clearly and accurately presented and does it cite the current literature?

Yes

If applicable, is the statistical analysis and its interpretation appropriate?

Yes

Are all the source data underlying the results available to ensure full reproducibility?

Yes

Are the conclusions drawn adequately supported by the results?

Partly

Are sufficient details of methods and analysis provided to allow replication by others?

Partly

Reviewer Expertise:

NA

We confirm that we have read this submission and believe that we have an appropriate level of expertise to confirm that it is of an acceptable scientific standard.

# Reviewer report: Reviewer response for version 1

**DOI:** 10.21956/openreseurope.14986.r28381

**Published:** 2022-02-04

**Authors:** Michael Carl, Longhui Zou

**Affiliations:** 1 Kent State University, Kent, OH, USA

This paper investigates effects of syntactic priming for L2 learner using Google Neuro Machine Translation (GNMT). It addresses several topics including implicit language learning, process of syntactic priming, human-computer interaction (HCI), computer assisted second language learning (SLA). The introduIdction and background give an informative overview of current research on syntactic priming in the fields of HCI and SLA.  Different from previous studies, this paper investigates the cumulative effect of syntactic priming on SLA via GNMT output.  

 The paper presents an empirical study with a total of 30 participants (native Brazilian Portuguese, L2 English speakers) translate a Portuguese prepositional noun phrase (PNP) into English. Portuguese only allows for the PNP structure, while in English it can be expressed by either PNP: “The *window of the office* is broken”, or a noun phrase structure, NP: “The *office window* is broken”. It is assumed that the less proficient English L2 speakers will more often (re)produce the PNP structure in English in an unprimed pre-test phase of the study’, but when primed with the GNMT output (which produces more often NP than PNP) they would learn and prefer the more common English NP rendering. Using a “logistic mixed effects maximal model”, the results show that, indeed, NP structures were more frequently used by the participants in the priming phase than the pre-test phase. The authors conclude that GNMT can facilitate the processing of more challenging structures in the second language and can trigger implicit learning of a second language by syntactic priming effect. The study also finds that implicit learning occurs at both lower levels and more advanced levels of English proficiency.

 Overall, the methods, materials and procedures of the experiment are clearly illustrated, and the statistical analysis are in sufficient details, which can largely benefit replication and future studies.

 However, there are still certain space for improvement:

- The study investigates the learning process of challenging syntactic structures for L2 English learners with GNMT, but there is inadequate literature review or empirical evidence to support that NP production is more challenging or complex to the participants than PNP production in their L2.
- On page 7, the author mentions that one of the inclusion criteria of the participants was “to be at intermediate or advanced English levels”, but the participants’ English proficiency levels were tested after completing both the pre-test and priming test phases. This sequence of experimental design seems unclear to me.
- The authors state that in their manual coding, “we coded the baseline as ”1” if participants produced a NP structure, suggesting they were primed by the GT output and ”0” if participants produced a PNP structure”. According to my understanding of the research procedures, in the baseline phase, the participants did not use GNMT, so this paragraph is somewhat confusing.
- I have some concern about the priming test phase, during which the participants were asked to translate the prime sentences using GT application on their own mobile device. There might be certain possibilities that the MT output of the same priming sentence would be different across the 30 participants during the experiment, which is undesirable and should be controlled.

It is unclear what exactly the study wants to test. The RQ1: “Can Google translate facilitate the processing of more challenging structures in the second language?” is somewhat not really a ‘research’ question, given that we know google translates every day billons (or so) words (and structures) for millions of users, who would otherwise not be able to process the second language at all. When reading, the paper appears to investigate: 1) the syntactic priming effect when translating Portuguese PNP or 2) how much is MT helpful for L2 learners. The paper provides plenty of evidence for syntactic priming effects (i.e. 1) from previous research, but, unfortunately does not address 2) very much (where it is not identical to 1). Only in the conclusion the authors argue that “regardless of the easily observable gaps in the translation quality of raw MT output, speakers trust Google translate enough to use the same syntactic structure seen in the MT output in their speech and learn from it”. Ah!

- it should be made clearer, from the beginning, that the texts were spoken. Would they speak the texts into their mobile phones? Was the prime also an auditory signal?
- It might be interesting to quantify MT quality and include the variable in the data analysis part, to investigate whether the difference in MT quality of the priming sentences would affect the NP production or SLA.
- A quite different experimental set-up should be used to assess ‘trust’ in the MT output, with different MT systems, different baselines, and maybe also translationese.

While the study shows “that the GNMT system influences the processing of English syntax”, it is unclear to what extent this is due to the particular brand, how MT errors impact the learning effect, and what effect might have the mode (spoken vs. written translation).

 Minor issues:

- Use only one of the two acronyms GT or GNMT.
- Figures do not show the pictures.

Is the study design appropriate and does the work have academic merit?

Partly

Is the work clearly and accurately presented and does it cite the current literature?

Yes

If applicable, is the statistical analysis and its interpretation appropriate?

Yes

Are all the source data underlying the results available to ensure full reproducibility?

Yes

Are the conclusions drawn adequately supported by the results?

Partly

Are sufficient details of methods and analysis provided to allow replication by others?

Partly

Reviewer Expertise:

NA

We confirm that we have read this submission and believe that we have an appropriate level of expertise to confirm that it is of an acceptable scientific standard, however we have significant reservations, as outlined above.

# Response

**Published:** 2023-12-04

**Authors:** NATALIA RESENDE

First, I want to express my sincere gratitude for the thorough review of this paper, and I truly value all the insightful comments that have been provided. Your feedback has proven to be immensely valuable in enhancing the overall quality of the paper. In my response to your comments, I have diligently addressed each one individually. Please find below my responses to each of the comments : 

 •    The study investigates the learning process of challenging syntactic structures for L2 English learners with GNMT, but there is inadequate literature review or empirical evidence to support that NP production is more challenging or complex to the participants than PNP production in their L2. Thank you for bringing up this point. I agree that the paper did not provide specific evidence supporting the claim that NP (noun phrase) production is more challenging or complex for the participants in their L2 (second language) compared to PNP (post-nominal possessive) production. As far as I'm aware, there is no existing evidence to support this assertion.

 However, in the revised version of the paper, I have addressed this issue by expanding a paragraph where I explain that NP production is considered more challenging for BP (Brazilian Portuguese) speakers. This assertion is based on previous studies conducted for other languages, which have shown that when a syntactic structure in the L2 does not exist in the L1 but there is a syntactic alternative in the L2 that closely matches the L1 structure, speakers tend to choose the one that is closer to the L1 structure. This preference arises because it represents an easier association due to L1 transfer processes.

 The expanded paragraph is provided below:  In Portuguese, only one syntactic alternative exists to represent a relationship of possession between nouns. The relationship is always encoded in the preposition do (de + o) or da (de + a) (e.g. a mesa do escritório está cheia or a porta da casa está fechada). However, in English, this relationship can be represented using either a prepositional noun phrase (PNP), which follows the same word order as in Portuguese (e.g. the table of the office is full) or a non-prepositional noun phrase (NP) (e.g. the office table is full), which differs from Portuguese in word order. Given that NP structures do not exist in Portuguese, we posit that they pose more difficulty for native Brazilian Portuguese speakers. As a result, these speakers might produce more PNP sentences due to their similarity with Portuguese syntax that may be transferred to their second language. While no research indicates that producing NP structures in English is more difficult for Brazilian Portuguese speakers than PNP, we assumed it is a more challenging structure because some studies (e.g. Vilela et al, 2010, Zara et al., 2013) with Brazilian Portuguese speakers of English as L2 have shown that when a syntactic structure in the L2 does not exist in the L1 but there is in the L2 a syntactic alternative that matches the L1 structure speakers tend to choose the one that is closer to the L1 structure because it is an easier association due to L1 transfer processes. Specifically for the syntactic structure to express a relation of possession, the study from Ghilzai (2014) shows that, when rating preferred English sentences with possessive structures, German speakers of English as L2 often rate higher the sentences using the PNP (of-genitive)  with an animate subject instead of the s-genitive case because, differently from English, the s-genitive in German is only used with proper nouns. Therefore this study is another evidence for transfer trends interfering in the L2 process. Hence, studying how Brazilian Portuguese speakers process these structures in English can help determine if syntactic priming by the GNMT output prompts them to use the NP format more frequently in English. •    On page 7, the author mentions that one of the inclusion criteria of the participants was “to be at intermediate or advanced English levels”, but the participants’ English proficiency levels were tested after completing both the pre-test and priming test phases. This sequence of experimental design seems unclear to me. I clarify this in the paper as can be read below:  We analysed data from 30 volunteer Brazilian Portuguese L2 English speakers (10 men, mean age=35.7 - sd=5.3). Participants were recruited through posts on Facebook groups of Brazilians living in Dublin and word-of-mouth recommendations. All participants were requested to read a plain language statement and sign the informed consent form to take part in the experiment. The inclusion criteria to take part in the study was to be a native speaker of Brazilian Portuguese, to live in Dublin at the time of the data collection, to have used GNMT as a tool supporting spoken English and be currently enrolled or have previously attended an English school in Dublin, with English proficiency at either intermediate or advanced levels. If not actively enrolled, participants had to share their most recent proficiency level upon exiting the English school. In order to confirm participants’ English proficiency levels we asked them to complete the General English test online with 25 questions immediately after completing the pre-test and priming test phases. Participants were classified at the basic level (level A2) with scores ranging from 2 to 13; pre-intermediate level (B1) scores from 14 to 17; intermediate level (B2) scores from 18 to 19; advanced level scores from 20 to 22 and proficient level scores from 23 to 25. Participants reporting an intermediate level from their English school but scoring at the basic level on our test were excluded. The mean score of the participants included in the experiment was 14.1 (SD=4.7) or level B1). In return for taking part in the experiment, all participants received a €10 voucher.  

 •    The authors state that in their manual coding, “we coded the baseline as ”1” if participants produced a NP structure, suggesting they were primed by the GT output and ”0” if participants produced a PNP structure”. According to my understanding of the research procedures, in the baseline phase, the participants did not use GNMT, so this paragraph is somewhat confusing. Thanks for pointing out this oversight of my part. I have reviewed this paragraph by removing the sentence  “suggesting they were primed by the GT output” Reviewed version: Participants’ responses for both baseline and priming tests were manually coded by the experimenter. In the baseline phase, responses with an NP structure were coded as "1", while those with a PNP structure received a "0". For the priming phase, if participants used the same structure in the target item as seen in the GNMT output (specifically, NP structures) to describe the images, it was coded as "yes" (1). If they used a PNP structure or another variant like "the office’s window is broken" or "the window in the office is broken", it was coded as "no" (0).  

 •    I have some concern about the priming test phase, during which the participants were asked to translate the prime sentences using GT application on their own mobile device. There might be certain possibilities that the MT output of the same priming sentence would be different across the 30 participants during the experiment, which is undesirable and should be controlled.

 Yes, I was aware that some variation in the output of GT could occur. In the first version,  I mistakenly did not add a paragraph in this article to explain what was the procedure to address this issue. However, this issue was addressed in this new version as below: It is important to note that when we created the prime sentences, they had all been machine-translated using GNMT on various days and different times spanning two months. We observed that the sentence structures remained consistent over time as Google consistently translated all sentences using an NP structure. We were aware, however, that despite this consistency over time, GNMT output could still vary across experiment trials during sessions. However, because we noticed that GNMT’s preferred structure is an NP structure, we thought it would be worth testing whether GNMT output would be capable of eliciting priming effects even if in a few cases the system outputs an alternative structure. It is unclear what exactly the study wants to test. The RQ1: “Can Google translate facilitate the processing of more challenging structures in the second language?” is somewhat not really a ‘research’ question, given that we know google translates every day billons (or so) words (and structures) for millions of users, who would otherwise not be able to process the second language at all. When reading, the paper appears to investigate: 1) the syntactic priming effect when translating Portuguese PNP or 2) how much is MT helpful for L2 learners. I reworded the RQ1 (see below). Hope it is clearer now: “When interacting with Google translate for language production purposes, can the output of Google translate facilitate the processing of more challenging structures in the second language?” 

  The paper provides plenty of evidence for syntactic priming effects (i.e. 1) from previous research, but, unfortunately does not address 2) very much (where it is not identical to 1). Only in the conclusion the authors argue that “regardless of the easily observable gaps in the translation quality of raw MT output, speakers trust Google translate enough to use the same syntactic structure seen in the MT output in their speech and learn from it”.  

 •    it should be made clearer, from the beginning, that the texts were spoken. Would they speak the texts into their mobile phones? Was the prime also an auditory signal?

 I improved the wording when describing the procedures to clarify that the prime sentences were presented in the written form but the translation was carried out verbally •    It might be interesting to quantify MT quality and include the variable in the data analysis part, to investigate whether the difference in MT quality of the priming sentences would affect the NP production or SLA. it's worth noting that throughout our experimental sessions, we consistently observed Google Translate using the NP (noun phrase) structure. Consequently, this particular variable didn't appear to have any noticeable impact on the results. In fact, it was precisely this consistency that gave rise to the observed priming effect.  •    A quite different experimental set-up should be used to assess ‘trust’ in the MT output, with different MT systems, different baselines, and maybe also translationese. 

 While the study shows “that the GNMT system influences the processing of English syntax”, it is unclear to what extent this is due to the particular brand, how MT errors impact the learning effect, and what effect might have the mode (spoken vs. written translation). 

 This is a good suggestion for future research. This is one of the questions that I want to address in future experiments.   

 Minor issues: •    Use only one of the two acronyms GT or GNMT.  Acronym issue fixed

 •    Figures do not show the pictures

# Reviewer report: Reviewer response for version 1

**DOI:** 10.21956/openreseurope.14986.r27633

**Published:** 2021-10-19

**Authors:** Cândido Oliveira

**Affiliations:** 1 Centro Federal de Educação Tecnológica de Minas Gerais, Belo Horizonte, Brazil

Thank you for the opportunity to review **Investigating syntactic priming cumulative effects in Mt** **-** **human** **interaction**. The study and its findings are very interesting. I hope the following feedback will provide helpful guidance in shaping the manuscript.

   

 The article presents a study that aims to investigate the interaction between Google neural machine translation system (GNMT) and English language learners whose first language is Brazilian Portuguese (BP). The author conducted a syntactic priming experiment with a pre-test-priming design to analyze whether the interaction with GNMT could lead these participants to use the structure that is the most preferred one among English native speakers to indicate a relation of possession (the book cover is red) more often than the structure whose equivalent in Portuguese is the most preferred one among BP native speakers (the cover of the book is red / *a capa do livro é vermelha*). The results indicate that participants (i) tended to use the former structure to form a sentence after they had just been exposed to it in a translation process (priming effect) and (ii) this tendency increased as they were continuously exposed to this structure (cumulative effect). The author suggest that these results indicate that GNMT can influence learners’ behavior in the L2 as well as their learning process.

 The text reads well and has a good organization. The references are relevant and up to date. The theoretical and applied motivations are clearly established. I found the justifications to investigate MT-human interaction through the use of google translate by language learners very interesting. Moreover, the methodology, especially the method, and well justified and described in great details. I have only three suggestions to the author, which I present below:

 On page four, the author presents two studies that seem to be part of the same project this article is inserted in. In order for the readers to have a better picture of these previous articles, I recommend the author includes information about the participants proficiency level. Also, it is not clear if there was any empirical evidence that participants indeed had a tendency to use the PNP instead of the NP structure (the present article does that) before the experiments were carried out.

 I was under the impression that the low number of distractors in the present study could have had an effect on the results. I suggest the author include a justification for the number of distractor items utilized in the experiment and/or a discussion about it. Other studies using priming, including some that were cited by the author (ex: Hartsuiker & Westenberg, 2000) use a much higher number of distractors.

 The use of psycholinguistic methods to bridge the gap between second language acquisition theories and language learning itself has been growing exponentially in recent years and this study adds to this process. Even though, it is not the focus of the study, I believe the author could include some discussions about the implications of the results to the field of Second Language Acquisition and to language learning in general.

Is the study design appropriate and does the work have academic merit?

Yes

Is the work clearly and accurately presented and does it cite the current literature?

Yes

If applicable, is the statistical analysis and its interpretation appropriate?

Yes

Are all the source data underlying the results available to ensure full reproducibility?

Yes

Are the conclusions drawn adequately supported by the results?

Yes

Are sufficient details of methods and analysis provided to allow replication by others?

Yes

Reviewer Expertise:

Bilingualism, Psycholinguistics and Second Language Acquisition

I confirm that I have read this submission and believe that I have an appropriate level of expertise to confirm that it is of an acceptable scientific standard.
